# On Chlorosis in Adults

**Published:** 1846-09

**Authors:** 


*On the Chlorosis of Adults.* By M. Blaud. There is a disease, pretty commonly met with, which seems to me to have escaped the observation of most practitioners. This affection, of great danger if misunderstood, is the *Chlorosis of Adults.* I do not allude to that decoloration of the skin dependent upon some prior disease, such as hemorrhage, intermittent fever, or some organic lesion, acute or chronic, &c.; but to an *essential idiopathic chlorosis*, proceeding from no other cause but such as produces it directly, that is, a particular defect of hæmatosis, and which can only be relieved by re-establishing the normal condition of this function.

Chlorosis is by no means peculiar to young girls arrived at puberty, and it is from want of attention to this fact, that many practitioners have mistaken it, when occurring in adults, for different organic lesions which in fact had no existence. Some, seeing the pale or yellow skin, have declared it to be symptomatic of hepatic lesion, and others have attributed it to affection of the spleen. Some attribute the dyspnœa and palpitations to lesions of the heart or large vessels, and others see in these the symptoms of an asthma. Such different views lead to various plans of treatment, none of which are efficacious, the chlorosis, indeed, seeming to derive a new intensity from their operation. Chlorosis may attack adults of either sex, any age, and of any condition in life. We have seen subjects of it, persons leading the most active and laborious lives, and others passing their time in luxurious idleness. The sober and the intemperate; those whom misery oppresses, and those who enjoy every abundance in life, may alike become its victims. So that the *predisposing* causes of the affection are as yet very obscure. The colorless and serous condition of the blood, the general debility, and languor of every function, seem to show that defective hæmatosis is the *proximate* cause. The *symptoms* are the same as in the chlorosis of young girls,—such as the yellowish-green skin (the conjunctivæ preserving their normal whiteness,) dyspnœa upon locomotion, *bruit de souffle* in the course of the carotids, &c. Still there are modifications noticeable. The color of the skin is rather greyish or earthy, than yellow, in consequence of its rough or wrinkled state, especially in the face. The palpitations are more severe; while there is moreover a deep, insupportable *malaise*, sometimes accompanied by a disposition to suicide, and a more or less abundant anal hæmorrhage coming on at irregular intervals. With the general langour and disorder of the digestive organs, there is œdema of the extremities; and, if the disease be left to itself, a serous effusion into the cavity of the abdomen results from the atony of the absorbents. However severe these symptoms may appear, they never resist appropriate *treatment*, when this is not resorted to too late.

Of the cases he has met with, M. Blaud selects eight for description, four of them being women from 26 to 37 years of age, and the others, men engaged in active or laborious occupations. In most of these, various kinds of treatment had been employed before they came under the author's care, in the idea that organic disease was present: but he, guided by the general appearance of the patient and the absence of marked signs of such lesions, treated them all for chlorosis with complete success. He employed his own anti-chlorotic pills,* which have attained so high a reputation in France, in every case; and complete cures resulted in periods varying from 10 to 30 days of taking them. He adds in a note, that long experience has convinced him that these pills are also a specific in *intermittent fever accompanied with œdema of the lower extemities*, which has resisted quinine and all other febrifuges.—*Med. Chir. Rev.—Bul. Med. Sci.*

*Made thus: ℞ Equal parts of sulph. iron and carb. potas, powder separately and mix intimately; make into pills of six grains; of which 2 to be taken thrice daily for 3 days; 4 as often for as many days; and then 6 three times daily till cured.

